# Effect of Gamma Irradiation on Depolymerization and Property Changes of Gum Tragacanth

**DOI:** 10.1155/ijbm/8875341

**Published:** 2024-11-30

**Authors:** Boontiwa Ninchan, Parimitta Chauywongyart, Teerawat Utapong, Nuatawan Thamrongsiripak

**Affiliations:** ^1^Department of Biotechnology, Faculty of Agro-Industry, Kasetsart University, Bangkok 10900, Thailand; ^2^Sugars and Derivatives Analytical Laboratory, Department of Biotechnology, Faculty of Agro-Industry, Kasetsart University, Bangkok 10900, Thailand; ^3^Irradiation Center, Thailand Institute of Nuclear Technology (Public Organization), Nakhon Nayok 26120, Thailand

**Keywords:** depolymerization, gamma radiation, gum tragacanth, irradiation, polysaccharides

## Abstract

High-energy nonthermal processes (irradiation) are an interesting technique for depolymerization. Gum tragacanth (GT) is a heteropolysaccharide composed of various sugars that are beneficial in the food and pharmaceutical industries. This study investigated the effects of different gamma irradiation doses (2.5, 5, 10, 20, 100, 500, 1,000, and 2000 kGy) on GT properties, considering both structural and physicochemical changes. The results confirmed that gamma irradiation influenced depolymerization with increases in monosaccharides (L-arabinose, D-galactose, D-glucose, D-xylose, L-fucose, L-rhamnose) and the percentage of degradation. Fourier transform infrared (FTIR) spectroscopy analysis indicated that structural changes occurred, with more free O-H and C-O bonding, including the carboxylic group (COOH) in the degraded molecules after irradiation. The changes in physicochemical properties were lower viscosity and a color change under gamma irradiation. The property changes in the GT were clearly related to an increased dose of gamma rays. In summary, there was comprehensive GT degradation following exposure using different increasing doses of gamma radiation, with some concomitant property changes in the GT.

## 1. Introduction

A natural gum is generally composed of various compositions such as monosaccharides, oligosaccharides, polymers, organic acids, etc., as same the composition of gum tragacanth (GT). Some compositions of natural gum are interesting and are benefits that can be applied in many applications and many industries. How to extract these compositions? Then, the potential degradation method is important and first priority on research topic of natural gum degradation. Moreover, the efficient extraction and purification after gum degradation also are the interesting further research studies.

GT is an exudate complex consisting of a highly branched heteropolysaccharide hydrocolloid extracted from the plants of the genus *Astragalus* [1, 2]. GT has been accepted as generally recognized as a safe (GRAS) by the Food and Drug Administration (FDA) [2, 3]. Consequently, it is widely used in food applications and in the pharmacological industry with its properties making its predominant use as an emulsifying/suspending agent. In addition, GT is used in healthcare-related products and in biomedicine applications due to its potential biocompatibility properties in hard and soft tissue engineering, skin regeneration, and wound healing [3]. Structurally, GT is composed of heteropolysaccharides containing complex sugars and uronic acid units, such as L-arabinose, D-galactose, D-glucose, D-xylose, L-fucose, L-rhamnose, and D-galacturonic acid that are linked together as polymers, such as arabinogalactan and xylogalacturonans [1, 4]. Terminal fucosyl residues in the form of fuco-xylo-substituted structures have also been identified in this gum [4]. L-fucose is mainly found in human breast milk in the form of derivatives of human milk oligosaccharides (HMOs) that predominantly support health-promoting activities [5, 6]. Different species of *Astragalus* have been reported to contain different ratios of components [7] that in GT are present as beneficial monosaccharides that could have any useful applications.

Gamma irradiation is an ionic and nonthermal process using gamma rays that are very high energy and can break the chemical bonds in materials [8] and degrade the polymeric structure of carbohydrate [9, 10]. Thus, irradiation can change the structure and some physical and rheological properties of polysaccharides as well as their end products, such as color, pH, viscosity, solubility, and molecular weight [11]. Moreover, irradiation is non-chemical process that is eco-friendly degradation method. Nowadays, the irradiation application is widely used in many industries such as food irradiation for inhibition of sprouting of agricultural crops, food ingredients, and ready-to-eat food products as the food sterilization process [8]. Moreover, the packaged products can control or inhibit the spoilage from microorganisms by irradiation. These are the important reasons using gamma irradiation on this research.

The current study investigated the effect of gamma irradiation on GT properties based on structural changes, such as the percentage of degradation, monosaccharide composition, the structure of the degraded polymer, and physicochemical changes, such as the color and viscosity properties of the gum polysaccharide after irradiation. The results should provide comprehensive information on GT degradation from using different doses of gamma radiation and some of the subsequent property changes to the GT. Moreover, the expected findings can reveal an alternative efficient method for gum depolymerization of GT using gamma irradiation.

## 2. Materials and Methods

### 2.1. Materials

GT was purchased from Loba Chemie Pvt. Ltd. (India). All chemicals were analytical grade. The standard sugars (D-glucose, D-xylose, D-galactose, L-rhamnose, L-arabinose, and L-fucose) were supplied by Toronto Research Chemicals Inc. (Canada).

### 2.2. Gamma Irradiation on GT

Samples of GT powder were irradiated at 2.5, 5, 10, 20, 100, 500, 1,000, or 2000 kGy with a fixed dose rate of 4.46 kGy/hr from a cobalt-60 gamma irradiator using the multipurpose gamma irradiator (Paul Stephens Consultancy Ltd, UK). The container for irradiation was a carrier with a diameter of 30 cm and a height of 15 cm, with the biological shielding consisting of a 1.88 m thick concrete wall. The irradiated samples were stored in sealed bags at room temperature. Irradiation was performed at the Thailand Institute of Nuclear Technology (Public Organization), Ongkharak, Thailand.

### 2.3. Determination of Percentage Degradation Under Different Gamma Ray Doses

GT is a heteropolysaccharide composed of sugars. The total sugar content in the nonirradiated GT (control) was determined using the phenol-sulfuric acid method [12]. The efficiency of gamma irradiation increased the breakdown into reducing sugar molecules, with the reducing sugar analyzed using the Somogyi–Nelson method [13]. The percentage of degradation was estimated by the ratio of reducing sugar released-to-total sugars, using equation [14]:

(1) $$\begin{array}{c} \text{Degradation }\left(\%\right)=\frac{\text{Reducing sugar released }\left(\text{Final}-\text{Initial sugar}\right)}{\text{Total sugar content}-\text{Initial reducing sugar }}\times 100. \end{array}$$

### 2.4. Compositional Analysis of Sugars and Polysaccharides Using High Performance Liquid Chromatography (HPLC)

The type and amount of each sugar were analyzed following Ninchan and Noidee [15, 16] and Vertical Chromatography Co., Ltd. [17]. All samples (non-irradiated and irradiated gum) were diluted to the appropriate level of concentration and filtered through a cellulose acetate membrane with a diameter of 0.45 *μ*m. Thereafter, analysis of sugars content was performed based on HPLC (Shimadzu Corporation; Japan) and measured using a refractive index detector (RID-10A; Shimadzu Corporation; Japan) with VertiSep^TM^ Sugar LMP column (7.8 × 300 mm, 8 *μ*m; Vertical Chromatography Co., Ltd.; Thailand) at 80°C, using deionized water as the mobile phase at a flow rate of 0.4 mL/min with standard sugars (D-glucose, D-xylose, D-galactose, L-rhamnose, L-arabinose, and L-fucose) as monosaccharides to study the depolymerization due to gamma degradation.

### 2.5. Fourier Transform Infrared (FTIR) Spectral Analysis

FTIR was recorded on a Bruker spectrometer (Model Tensor 27; Germany) in the range 400–4000 cm, at a resolution of 4 cm^−1^ and 64 scans using the KBr-pellet method. Briefly, the GT samples were pressed directly on an attenuated reflectance KBr tablet (KBr was supplied with the FTIR unit) into the sampling unit. Infrared measurement was carried out in transmission mode, in which the infrared beam passed directly through the sample and was then recorded in the transmittance unit. The infrared spectra were presented using Star Software version 16.30 a.

### 2.6. Measurement of Viscosity

Non-irradiated and irradiated gum samples were prepared at 5% w/v and their viscosity was measured using a Brookfield Visco meter (model DV2T Viscometer; Brookfield Baths; USA). A rate of 25 rpm was applied at 25 ± 0.5°C for 5 min using an LV-04 spindle (no. 64). All samples were measured in three replicates.

### 2.7. Color Determination

The index based on the *L*^∗^, *a*^∗^, and *b*^∗^ values was determined for each nonirradiated and irradiated gum powder sample using a spectro-colorimeter (Ultrascan PRO; Hunter Lab; USA) using reflectance specular excluded (RSEX) mode (0.780 UVF nominal with a specular excluded large area view (LAV) aperture size of 25 mm). The instrument was used with d/8° geometry and a 6-inch integrating sphere coated with Spectraflect, and a port plate and specular exclusion port door coated with Duraflect (57% relative humidity and 27°C). The results were expressed in accordance with the CIELAB system with reference to illuminant/visual angle observer D65/10°. The CIE system uses three terms (*L*^∗^, *a*^∗^, and *b*^∗^) to represent color, where *L*^∗^ represents lightness, with values from 0 (black) to 100 (white), *a*^∗^ represents red with a positive value and green with a negative value (green), and *b*^∗^ represents yellow with a positive value and blue with a negative value. All measurements were performed in triplicate and the results were averaged.

### 2.8. Statistical Analysis

Analytical values were analyzed and reported as the mean and standard deviation. All reported values were based on the mean of three replicates. Analysis of variance (ANOVA) was used for data analysis in the IBM SPSS Statistics Version: 29.0.0.0 (241) software. Significance was tested at the *p* < 0.05 level in the ANOVA.

## 3. Results and Discussion

### 3.1. GT Degradation Under Different Gamma Ray Doses

The heteropolysaccharide chemical structure of GT was degraded by the gamma rays into short chains of polysaccharides and some monosaccharides that were determined in terms of reducing sugar content. However, the non-irradiated GT (control) is normally composed of trace amounts of the reducing sugars (Table 1). The increasing reducing sugar content indicated the efficiency of the high energy of gamma ray to degrade the GT structure. As shown in Table 1, the gamma ray dose was positively correlated with the reducing sugar content and percentage of degradation, with levels below 2.5–20 kGy having no significant effect on the efficiency of degradation (less than 1% degradation). However, a gamma dose of more than 100 kGy had a highly significant impact on the efficiency of degradation with high percentages of degradation and the reducing sugar content. The maximum GT hydrolysis was 25.93% with the highest gamma dose at 2000 kGy.

### 3.2. Sugars and Polysaccharides Composition

**Figure 1:**
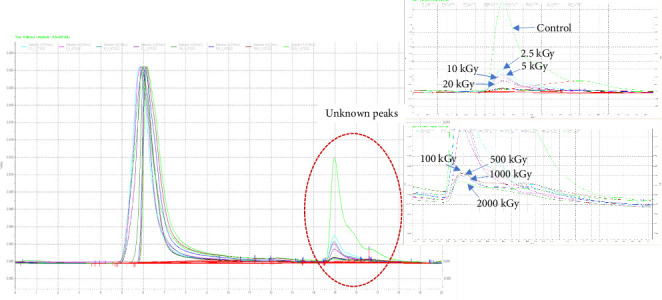
HPLC analysis of nonirradiated and irradiated gum using different gamma doses.

All sugars (glucose, xylose, galactose, rhamnose, arabinose, and fucose) have been reported as monosaccharides in the GT [1]. A higher dosage of gamma rays directly increased the degradation of monosaccharides. The results confirmed depolymerization under irradiation as indicated by the increases in the reducing sugar content and percentage of degradation (Table 1). However, some monosaccharides (xylose, galactose, and rhamnose) were not separated completely and so these were aggregated, as shown in Table 2. Their sum was positively correlated with the gamma dose applied, especially for more than 500 kGy which also produced significant increases in the arabinose and fucose contents (Table 2). Notably, the results from the HPLC analysis (Figure 1) showed clear unknown peaks not consistent with sugars and polysaccharides and that were not degraded monosaccharides. The characteristics of these unknown peaks declined clearly with increasing gamma dose, implying the depolymerization of xylogalacturonan or/and arabinogalactan, which are the main components in GT [1, 4]. Furthermore, the results showed increases in xylose, galactose, and arabinose, as shown in Table 2.

### 3.3. FTIR Spectra

**Figure 2:**
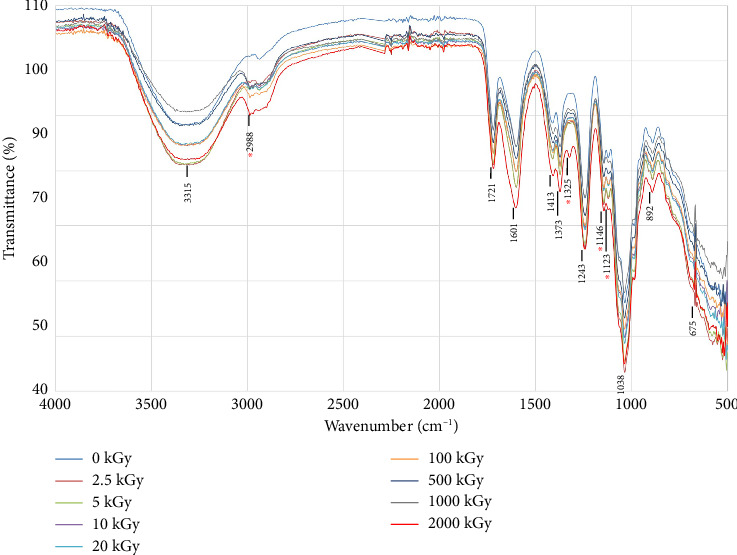
FTIR spectra of nonirradiated and irradiated gum after gamma irradiation.

The FTIR spectra of the non-irradiated and irradiated gum samples were compared based on the transmittance peaks (Figure 2). All spectra had quite similar absorption patterns as indicated by absorption bands that referred to C-H bending (500–880 cm^−1^), C=C bending (892 cm^−1^), C-O stretching (1038, 1243 cm^−1^), O-H bending (1325–1413 cm^−1^) and O-H stretching (2550–3300 cm^−1^) of alcohol and carboxylic group (COOH), C=C stretching (1601 cm^−1^), and C=O stretching (1721 cm^−1^). Some absorption bands showed a significant difference at 1123–1146 cm^−1^ which referred to C-O stretching, at 1310–1390 cm^−1^ which referred to O-H bending, and at 2500–3300 cm^−1^ which referred to O-H stretching of carboxylic acid.

The absorption of O-H group is observed at 2500–3300 cm^−1^ and 3200–3700 cm^−1^ in the functional group region (1500–4000 cm^−1^) that referred to O-H stretching of alcohol and carboxylic acid group. However, the absorption of O-H group can be observed at 1325–1440 cm^−1^ in the fingerprint region (500–1500 cm^−1^) that referred to O-H bending of alcohol and carboxylic acid group. In theory, the different spectrum at the same wave number especially in the fingerprint region can imply to the different structure of molecule. In Figure 2, the spectrums at 1325 cm^−1^ showed the different spectrums especially under gamma does at 2000 kGy. The highest does of gamma ray could degrade the gum polymer into smaller molecules resulting to increase free O-H group including the degraded molecules were probably different structures that related to increasing amounts of different sugars as shown in Table 2.

These bands were clearly evident with increasing doses of gamma rays due to the greater vibration and absorption of free O-H, C-O bonding of molecules, including the COOH due to degradation after the irradiation.

### 3.4. Viscosity Characterization

**Figure 3:**
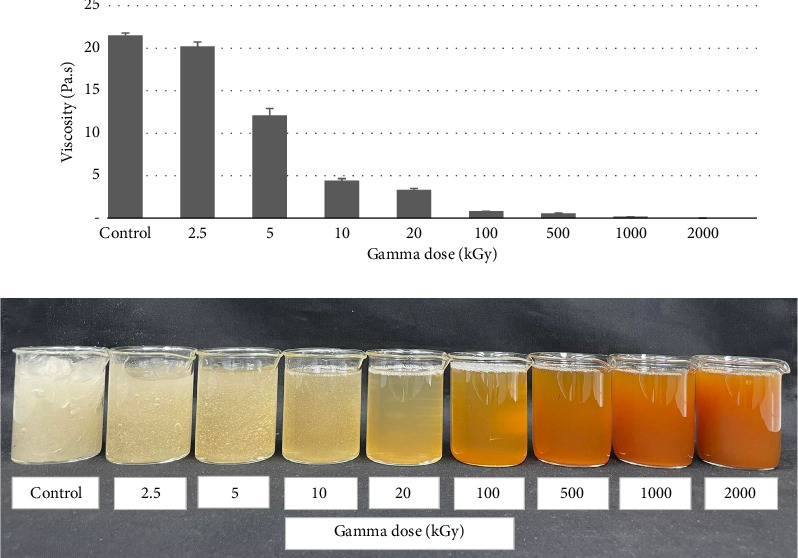
Viscosity and appearance of gum solution after gamma irradiation.

Generally, the size or molecular weight of the substance positively correlates with the viscosity property [18]. Polysaccharides with high molecular weight obviously show high viscosity. Depolymerization or the molecular weight of the substance is reduced, significantly decreasing viscosity [19]. The non-irradiated GT (control) is a complex polysaccharide or polymer that acts a viscous hydrocolloid with 21.51 Pa.s. The high energy of the gamma rays can potentially degrade the linkages of the GT complex molecules into smaller molecules, resulting in a decrease in the GT viscosity, as shown in Figure 3. The GT viscosity clearly decreased when the GT gum was degraded under higher gamma dose levels, with a significant, negative correlation. Furthermore, the appearance of all irradiated GT solutions changed to liquid-like with higher doses of gamma degradation. The change in color is explained and discussed below under color determination.

### 3.5. Color Determination

**Figure 4:**
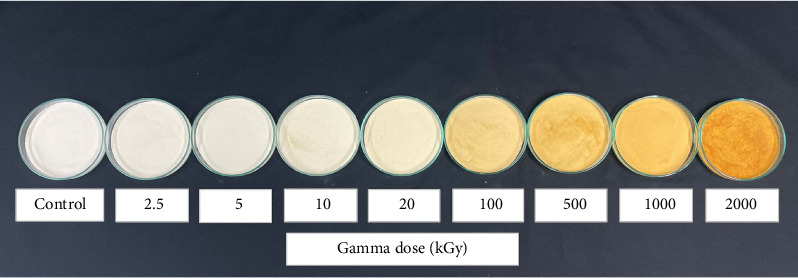
Color of GT powder after gamma irradiation.

The color of the irradiated GT changed after irradiation. The values of *a*^∗^, *b*^∗^, and *L*^∗^ for the non-irradiated and irradiated GT samples are shown in Table 3 and the color appearance of the GT powders after irradiation compared with non-irradiated GT (control) is shown in Figure 4. The results showed positive *a*^∗^ values that corresponded with red and positive *b*^∗^ that corresponded with yellow, while *L*^∗^ represented lightness. *L*^∗^ scale values at nearly 100 represent more whiteness. In all irradiated treatments, the values of *a*^∗^ and *b*^∗^ increased with increasing doses of gamma rays, while *L*^∗^ decreased. With high gamma doses of more than 100 kGy, the irradiated GT treatments clearly changed color, especially *a*^∗^ (red) and *b*^∗^ (yellow), which significantly increased. From these results, it was concluded that gamma ray darkened the GT samples after irradiation (low *L*^∗^ values). Radiation-induced darkening of polysaccharides has been reported elsewhere [8].

## 4. Discussion

This research confirmed that gamma rays are high energy that can break the molecule structure. Absolutely, increasing gamma doses positively related to the efficiency degradation which was confirmed significantly by the results of increasing percentage of degradation, amount of reducing sugars, degraded monosaccharides and polymers, and decreased viscosity. However, the identification of polymer type was a limitation of this study including some monosaccharides (xylose, galactose, and rhamnose) that were not separated completely then could not be identified for each. The increasing gamma doses also increased thermal energy to imply the burning that obviously negatively affected on the color property. All results that showed the changes in structural and physicochemical properties of GT after gamma irradiation were important data to support the consideration on the suitable applications of degraded GT in various industries, doses of gamma rays for the degradation, and more research on the degraded GT for extraction and purification in the future.

## 5. Conclusion

The current results confirmed that gamma irradiation influenced depolymerization, with increases in monosaccharides and the percentage of degradation confirmed following GT depolymerization. Structural changes were identified using FTIR analysis that showed free O-H and C-O bonding, including the COOH of the degraded molecules after irradiation. In addition, the physicochemical properties changed with lower viscosity and a darker color following gamma irradiation. Increasing the dose of gamma rays clearly changed the properties of the GT. Consequently, the application of gamma irradiation to a target material should be considered where the objective is to change the properties after irradiation. Moreover, some monosaccharides and degraded compositions were interesting for the further research studies on the study of extraction and purification including studies on the applications in the food and pharmaceutical industries.

## Figures and Tables

**Table 1:** Percentage of reducing sugar and degradation of gum irradiation using different gamma doses.

Gamma dose (kGy)	Reducing sugar (%)	Degradation (%)
Control	0.24 ± 0.00^e^	0.00 ± 0.00^e^
2.5	0.25 ± 0.00^e^	0.01 ± 0.00^e^
5	0.27 ± 0.00^e^	0.06 ± 0.00^e^
10	0.30 ± 0.00^e^	0.12 ± 0.01^e^
20	0.36 ± 0.00^e^	0.26 ± 0.01^e^
100	0.71 ± 0.01^d^	1.07 ± 0.02^d^
500	8.10 ± 0.24^c^	18.19 ± 0.56^c^
1000	10.04 ± 0.09^b^	22.66 ± 0.21^b^
2000	11.44 ± 0.23^a^	25.93 ± 0.54^a^

*Note:* Mean ± SD values with different lowercase superscripts in same column are significantly different (*p* < 0.05).

**Table 2:** Contents of free monosaccharides after gamma irradiation using different gamma doses.

Gamma dose (kGy)	Monosaccharide content (%)			
	Glucose	Xylose + galactose + rhamnose	Arabinose	Fucose
Control	2.27 ± 0.08^c^	0.58 ± 0.01^d^	0.34 ± 0.05^d^	0.035 ± 0.000^bc^
2.5	3.23 ± 0.02^b^	0.71 ± 0.00^d^	0.46 ± 0.05^d^	0.035 ± 0.001^bc^
5	3.66 ± 0.18^b^	0.89 ± 0.04^d^	0.66 ± 0.01^cd^	0.035 ± 0.000^c^
10	3.71 ± 0.06^b^	1.28 ± 0.03^d^	0.74 ± 0.05^cd^	0.035 ± 0.001^bc^
20	3.72 ± 0.00^b^	1.68 ± 0.14^d^	0.90 ± 0.01^cd^	0.035 ± 0.004^c^
100	3.82 ± 0.01^b^	1.88 ± 0.17^d^	2.36 ± 0.15^cd^	0.037 ± 0.002^bc^
500	4.36 ± 0.12^a^	4.89 ± 0.17^c^	5.95 ± 0.31^bc^	0.039 ± 0.003^b^
1000	4.72 ± 0.10^a^	11.37 ± 0.29^b^	7.86 ± 0.29^b^	0.048 ± 0.003^a^
2000	4.95 ± 0.23^a^	25.54 ± 0.27^a^	10.25 ± 0.50^a^	0.049 ± 0.002^a^

*Note:* Mean ± SD values with different lowercase superscripts in same column are significantly different (*p* < 0.05).

**Table 3:** CIE color parameters (*L*^∗^, *a*^∗^, and *b*^∗^) of GT powder after gamma irradiation.

Gamma dose (kGy)	CIE color parameter		
	*a* ^∗^	*b* ^∗^	*L* ^∗^
Control	2.18 ± 0.02^h^	9.56 ± 0.03^i^	86.65 ± 0.07^a^
2.5	2.33 ± 0.04^gh^	11.48 ± 0.06^h^	85.62 ± 0.06^b^
5	2.42 ± 0.05^g^	12.48 ± 0.25^g^	85.36 ± 0.12^b^
10	2.69 ± 0.02^f^	14.74 ± 0.21^f^	84.43 ± 0.09^c^
20	3.13 ± 0.02^e^	17.35 ± 0.06^e^	83.26 ± 0.07^d^
100	6.13 ± 0.12^d^	26.69 ± 0.41^d^	77.31 ± 0.24^e^
500	7.75 ± 0.05^c^	30.57 ± 0.25^c^	74.22 ± 0.16^f^
1000	9.83 ± 0.11^b^	32.72 ± 0.34^b^	70.58 ± 0.11^g^
2000	12.62 ± 0.22^a^	34.17 ± 0.34^a^	64.32 ± 0.31^h^

*Note:* Mean ± SD values with different lowercase superscripts in same column are significantly different (*p* < 0.05).

## Data Availability

The data used to support the findings of this study are included within the article.
